# AI-based shape optimization of galloping micro-power generators: exploring the benefits of curved surfaces

**DOI:** 10.1038/s41598-024-51979-8

**Published:** 2024-01-18

**Authors:** Hussam Alhussein, Ahmed S. Dalaq, Mohammed Daqaq

**Affiliations:** 1 Division of Engineering, New York University Abu Dhabi, Abu Dhabi, UAE; 2 Department of Bioengineering, King Fahd University of Petroleum and Minerals, Dhahran, Kingdom of Saudi Arabia

**Keywords:** Wind energy, Mechanical engineering

## Abstract

The advent of flow micro-power generation has resparked the interest in researching the galloping instability with the objective of determining the shape of the bluff body that is most prone to galloping. Such shape, which is sought to maximize the efficacy of galloping micro-power generators (GMPGs), must possess a very low cut-in flow speed while achieving large-amplitude steady-state oscillations beyond it. Additionally, since GMPGs can operate in environments with fluctuating flow conditions, the optimal shape must also have a very short rise time to its steady-state amplitude. In this work, we utilize computational fluid dynamics in conjunction with machine learning to optimize the shape of the bluff body of GMPGs for both steady-state and transient performance. We investigate a continuum shape description which encapsulates most of the cases studied earlier in the literature. The continuum has a straight frontal and dorsal faces with varying lengths, and side faces described by surfaces of different curvatures. The optimization study reveals that a curved-trapezoidal bluff body with the highest side surface curvature and frontal-to-dorsal ratio is the perfect shape for steady flow conditions. On the other hand, a square profile with the highest side surface curvature is the ideal choice for highly-fluctuating flow conditions because of its shortest rise time. The theoretical findings are replicated experimentally using wind tunnel tests.

## Introduction

The past 2 decades have witnessed the emergence of a new class of miniaturized energy harvesters that target low-level power generation for niche applications, mainly involving the deployment of self-powered sensors and sensing networks in remote and inaccessible locations. Such devices, which are commonly referred to as micro-power generators, need to be scalable and capable of harnessing energy efficiently from the energy sources available in their environment. Among the different types of micro-power generators are those designed to harness the kinetic energy of a moving fluid by exploiting aeroelastic instabilities such as vortex-induced vibrations^1^, wake galloping^2^, and transverse galloping^3^. In such devices, the aeroelastic instability sets an elastic structure or an oscillator into large-amplitude motions that can then be effectively harnessed using a scalable electro-mechanical transduction element (piezoelectric, electrostatic, or electromagnetic), which converts the captured energy into electricity.

This paper focuses on micro-power generators that exploit the galloping instability to harness energy from a flow. We will refer to those devices as galloping micro-power generators, or GMPGs for brevity. GMPGs, and rightfully so, have occupied the vast majority of the open literature because their operation does not require frequency matching and because they are capable of producing large periodic oscillations over a wide range of flow speeds^4^.

**Figure 1:**
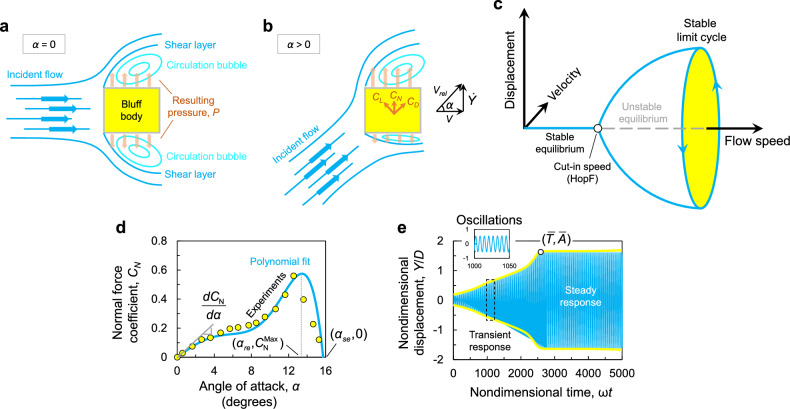
A schematic diagram of the galloping phenomenon showing the separation of shear layers and inner re-circulation bubbles responsible for the aeroelastic instability for two cases, where the angle of attacks (**a**) $\alpha =0$ and (**b**) $\alpha >0$. (**c**) Schematic showing the galloping bifurcation, including the cut-in speed (Hopf) and limit cycle oscillations. (**d**) Variation of the normal force coefficient, $C_N$ with the angle of attack, $\alpha$. The circular markers represent experimental data, while the solid line represents a polynomial approximation using $A_1 = 2.69$, $A_3 = -168$, $A_5 = 6270$, and $A_7 = 59{,}900$^5^. (**e**) Time history of the response showing the rise time $\bar{T}$ and the steady-state amplitude $\bar{A}$. The time, *t* is nondimensionalized by the natural frequency, $\omega$.

In general, galloping of an elastic structure occurs when an incident flow strikes a bluff body causing the flow to separate at the leading edge. As shown in Fig. 1a, the separation of the flow traps circulation bubbles between the shear layers and the side surfaces of the body. The characteristics of the circulation bubbles, such as their size and position relative to the body, vary with the angle of attack, $\alpha$. This variation induces a differential surface pressure, that in turn causes a resultant lift on the body (Fig. 1b). Now, if the energy channeled to the bluff body by the flow exceeds the energy dissipated by any damping mechanism, the amplitude of oscillations increases. This increase in amplitude further improves the asymmetry between the circulation regions, causing an even greater net lift. At a particular response amplitude, the direction of the net lift begins to oppose the velocity of the body, leading to fixed-amplitude periodic oscillations commonly coined as transverse galloping.

From a mathematical point of view, the galloping instability can be represented by a Hopf bifurcation point in the parameter space of the steady-state amplitude versus the flow speed (Fig. 1c). Below a critical flow speed called the cut-in speed, no steady-state galloping oscillations can be sustained, and the only possible steady-state response is the static equilibrium of the elastic oscillator. Beyond the cut-in speed, steady-state oscillations of a magnitude which increases with the flow speed can be incited.

It is well-known that the geometry of the “after-body” region plays an important role in the cut-in flow speed, and the amplitude of the resulting steady-state oscillations beyond it. This is primarily the case because the pressure loads associated with galloping act mainly on the after-body. Thus, prior to the advent of GMPGs, most research studies focused on investigating the relationship between the after-body shape and the lift and drag forces with the goal of mitigating or delaying this undesirable instability by increasing the cut-in flow speed. In one demonstration, Igarashi et al.^6^ investigated the galloping of a square prism at angles of attack ${0}^{\circ }<\alpha <{45}^{\circ }$, and showed that the angle at which the separated shear layers reattaches on either side of the bluff body strongly correlates with the maximum lift force. Using full field flow visualisation and force measurements on trapezoidal-section prisms, Luo et al.^7^ also revealed a direct correlation between the normal lift forces and the shear layers’ proximity to the after-body. Findings were also corroborated by other researchers using different bluff body shapes including rectangular^8^, circular^9^ and parabolic-based prisms^10^.

The advent of GMPGs has resparked the interest in researching galloping instability with the counter-objective of determining the bluff body shape, which is most prone to galloping; that is, the shape that has the lowest cut-in speed while achieving the largest steady-state oscillations beyond it. Among the well studied geometries (square, trapezoid, and D-section), Barrero et al. showed that the D-shaped prism is best suited for the design of GMPGs^11^. Yang et al.^12^, who carried out a theoretical and a numerical analysis of the performance of a piezoelectric GMPG, reported the superiority of the square cross-section. Ding et al.^13^ carried out two-dimensional numerical simulations of the galloping instability using four geometries: an equilateral triangle, a trapezoid, a circular cylinder, and a square. They reported that the best performing design in terms of flow energy extraction is the trapezoid. Recently, Barrero et al.^14^ also analyzed the impact of the shape of the cross-section on the energy extraction from transverse galloping and vortex-induced vibrations. Twelve different cross-sections were experimentally tested, and it was found that the D-section (half-cylinder) resulted in the best performance.

In addition to changing the shape of the afterbody, other researchers investigated the effect of adding a rigid attachment to the bluff body. In one demonstration, Noel et al.^15^ investigated the galloping response of various bluff bodies (square, trapezoid, and triangle) with an added horizontal tail-fin structure. They showed that, for some optimal fin lengths, the fin improves the steady-state amplitude of the response over the “fin-less” profiles. Subsequently, the same researchers introduced a Y-shaped tail fin instead of the horizontal one and reported that the performance of the GMPG’s can be dramatically improved for certain fork angles of the Y-fin^16,17^.

Despite the large body of research focusing on shape optimization of the bluff body for GMPGs, most of the prior studies have been limited by the following two factors:

- Research has focused on examining and comparing a few common body shapes without adopting a continuum approach to induce variations in the shape of the bluff body. This is because the process of modeling the fluid-structure interactions and estimating the aeroelastic forces require complex and computationally expensive models or a large number of costly and time consuming experimental tests.
- Research has focused on optimizing the shape of the GMPGs for steady wind conditions. That is, the performance and optimization analyses were based on the steady-state response curve of the GMPGs. However, unlike typical wind turbines, miniaturized GMPGs must be able to harness energy efficiently from wind gusts which usually fluctuate on a timescale that is slower than typical turbulence, but faster than a truly steady flow. To effectively capture energy in these environments, the harvester must be sensitive to flow fluctuations and have a short rise time to reach its steady-state value.

In this study, we address the above limitations by employing an approach that utilizes computational fluid dynamic (CFD) simulations combined with machine learning (see machine learning toolbox^18^) to optimize the shape of the bluff body of GMPGs for both steady-state and transient performance. We investigate a continuum shape description of a bluff body which has a straight frontal and dorsal faces with varying lengths, and side faces described by surfaces of different curvatures. As such, most of the cases discussed earlier in the literature (e.g. square, trapezoid and triangle) become special cases of our generalized shape description. Our objective here is to perform a full factorial study on a continuum of shapes with varied dorsal-to-frontal width ratios and radii of curvature of the side faces.

The machine learning algorithm is employed using regression artificial neural network (ANNs) as a surrogate for the CFD model^19,20^ (Supplementary Information, Fig. S1). Several recent works have employed this approach to speed up and assist optimization schemes^20–23^. However, the architecture of the ANN is often set arbitrarily or through trial-and-error. Here, the architecture of the ANN is parameterized and its hyperparamters are optimized for the problem at hand using genetic algorithms (GA)^24^ (see Supplementary Information, Fig. S2). Once the machine learning tool is optimized and is adequately trained through the computationally intensive CFD simulations, the surrogate model is used to evaluate unexplored regions within the design space, bounded indeed by the range of training data. Accordingly, the surrogate model allows rapid computation of the aeroelastic forces, whereby optimization over the design space can infer the optimum bluff body shape for GMPGs operating in steady and transient flow environments.

To this end, the paper is organized as follows: “Fundamental knowledge” establishes the fundamental understanding of the galloping instability and relates the characteristics of the quasi-steady normal force curve to the resulting galloping oscillations. “Geometric representation of the bluff body” presents the generic geometric description of the bluff body. “Optimization approach” layout the overall optimization steps followed in this study. “A high fidelity computational model” presents the high-fidelity computational model. “Artificial neural network” delves into the development and the optimization of the artificial neural network (ANN). “Results and discussion” delineates the effect of the body shape on the galloping response. “Flow field analysis” presents the flow field visualization around selected shapes. “Experimental results” presents experimental results for selected shapes. Finally, “Conclusion” presents the main conclusions. “Methods” are available at the end of the manuscript.

## Fundamental knowledge

Developing mathematical models to predict and understand the underlying dynamics of the galloping instability requires determining the aeroelastic forces acting on an oscillating body, which is a very complex task. To overcome this challenge, Parkinson proposed through several studies^5,25^ the quasi-steady theory, which until today remains one of the most widely-utilized, simple, yet relatively accurate approaches to model the galloping phenomenon. The quasi-steady theory states that the aerodynamic forces acting on a structure at each motion-induced angle of attack can be well-approximated by the static forces measured at the same angle of attack. This assumption is accurate as long as the time scale of the flow is significantly higher than that associated with the body oscillations, such that any unsteady perturbations in the flow are quickly carried downstream without influencing the motion of the oscillator. Using the aforementioned assumption, the governing dynamics of a bluff oscillator undergoing transverse motion, *Y*, can be approximated using the following ordinary differential equation^26^:

1 $$\begin{aligned} M \ddot{Y} + C \dot{Y} + K Y = \frac{1}{2} \rho H D V^2 C_{N}, \end{aligned}$$

where *M* is the mass of the bluff body, *C* is a linear damping coefficient, and *K* represents the stiffness. The first term on the left-hand side of Eq. (1) represents the inertial forces, the second term is a linear viscous damping force, the third term is used to model the restoring force element. The term on the right-hand side of Eq. (1) represents the aeroelastic normal force acting on the bluff body, where $\rho$ is the density of the flow, *D* is the characteristic width of the bluff body, *H* is the into-page depth of the body, *V* represents the flow speed, and $C_{N}$ is the normal force coefficient. Specific numerical values of spring stiffness, damping coefficient, mass and other geometric parameters can be found in Table 1 in “Methods” section.

**Table 1:** Material and geometric properties of the oscillator.

Property (symbol)	Value	Units
Mass (*M*)	0.45	$\hbox {kg}$
Damping (*C*)	0.025	$\hbox {N\,s}/\hbox {m}$
Linear stiffness (*K*)	360	$\hbox {N}/\hbox {m}$
Density ($\rho$)	1.225	$\hbox {kg}/\hbox {m}^3$
Frontal diameter (*D*)	0.035	$\hbox {m}$
Depth (*H*)	0.2	$\hbox {m}$
Natural Frequency ($\omega$)	28.2	$\hbox {rad}/\hbox {s}$

The quasi-steady assumption permits describing the normal force coefficient, $C_N$, as a function of the angle of attack, $\alpha$, by carrying out wind tunnel tests on a static bluff body. The process typically goes as follows: the bluff body is fixed in the wind tunnel at different angles with respect to the direction of a uniform flow of constant velocity, *V*. At each angle of attack, the normal force coefficient is measured. The curve is then fitted into a polynomial function of the angle of attack $\alpha$ using:

2 $$\begin{aligned} C_N = \sum _{j=1}^{n} A_{j} \alpha ^j, \end{aligned}$$

where $A_j$ are the aerodynamic force coefficients determined by fitting the experimental data into Eq.  (2), and *j* is an odd count for symmetric bluff body shapes. The normal force coefficient indicates that the aeroelastic force is dependent on the instantaneous angle of attack, $\alpha$. For small oscillations, the angle of attack can be approximated using Fig. 1b as

3 $$\begin{aligned} \alpha (\dot{Y}) = \frac{\dot{Y}}{V}. \end{aligned}$$

For a square prism, Parkinson et al.^5^ measured experimentally the $C_{N}$ curve as a function of $\alpha$ in static wind tunnel tests, and the resulting data points are shown in Fig. 1d as yellow circular markers with black outline. Time history of the response is obtained by integrating Eq.  (1) for the parameters shown in Table 1, a flow speed $V = 10$ m/s, and using the aerodynamic coefficients of the square obtained by fitting the experimental data into a polynomial (see solid blue line on Fig. 1d). The corresponding time history shows the rise of the response amplitude to the steady-state value, $\bar{A}$, and the rise time, $\bar{T}$ required to achieve it (Fig. 1e). A GMPG of excellent performance must have a low cut-in flow speed, a large steady-state amplitude at a given flow speed, and the shortest possible rise time to respond swiftly to flow fluctuations. These parameters are a direct function of profile of the lift curve which is unique for every geometry. Thus, optimizing the shape of the bluff body entails optimizing the shape of the lift curve.

**Figure 2:**
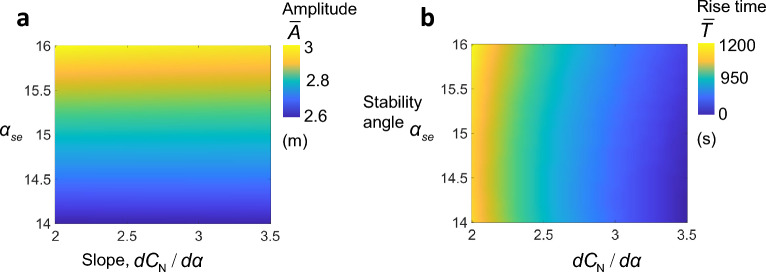
(**a**) Steady-state amplitude, $\bar{A}$ and (**b**) rise time $\bar{T}$ maps with variations in the stability angle and initial slope; $(\alpha _{se}, \frac{dC_N}{d\alpha })$.

The normal force curve has four important characteristics that determine the nature of galloping instability. These are the slope near $\alpha =0$, the angle at which the $C_N$ curve peaks, commonly known as the re-attachment angle^7^; ($\alpha _{re},C_N^{max}$) and the angle at which the curve re-crosses zero, that is the stability angle; $(\alpha _{se},C_N=0)$. To investigate how these characteristics influence the dynamic behavior of the galloping oscillator, we study their influence on the steady-state amplitude, $\bar{A}$, and on the rise time, $\bar{T}$. Numerical integration of Eq. (1) is carried for hypothetical curves of seventh-order polynomial as in Parkinson et al.^5^ (recall Fig. 1d). While the peak $C_N^{max}$ and the re-attachement angle $\alpha _{re}$ were fixed at 0.5 and ${12}^{\circ }$, the stability angle is varied between ${14}^{\circ }$ and ${16}^{\circ }$, and the initial slope, $dC_N/d\alpha$ ranges between 2 and 3.5. Figure 2a, shows that the steady-state amplitude is largely determined by the stability angle $\alpha _{se}$, where higher stability angles lead to higher $\bar{A}$. Furthermore, $\bar{A}$ is insensitive to variations in $dC_N/d\alpha$. Furthermore, higher $dC_N/d\alpha$ leads to shorter rise time, $\bar{T}$, to steady-state response. $\bar{T}$ is also insensitive to variations in the stability angle (Fig. 2b). Lastly, we exclude the peak $C_N^{max}$ and the re-attachment angle, $\alpha _{re}$ from the analysis because they have negligible effects on $\bar{A}$ and $\bar{T}$.

## Geometric representation of the bluff body

**Figure 3:**
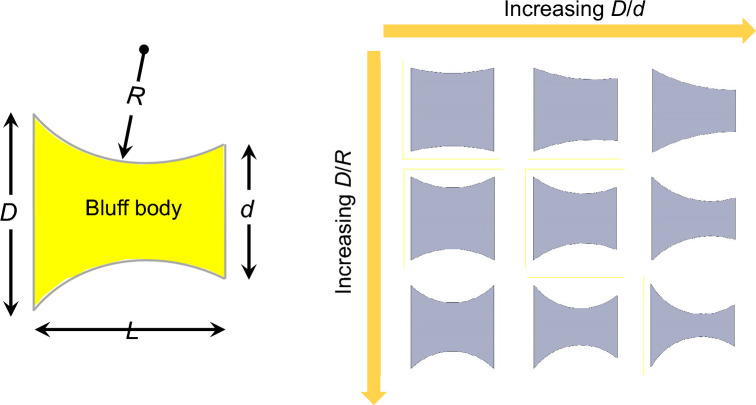
Schematic diagram of a curved-trapezoid cross-section, along with the continuum bluff body geometries with varying dorsal-to-frontal width ratio *D*/*d*, and radius of curvature *D*/*R*.

The current study examines the impact of afterbody shape on the performance of GMPGs. The continuum of geometries under examination possesses the same stream-wise length, *L*, and frontal width, *D*, as well as identical flow separation locations initiated at the frontal sharp edges. Figure 3 shows the family of shapes under consideration which has two defining characteristics:

- A gradual reduction in the dorsal width, *d*. In this study, the side ratio, *D*/*d*, is limited to the range between 1 and 1.66, because our investigation has revealed that when $D/d > 1.66$, the initial slope becomes; $\frac{dC_N}{d\alpha } < 0$. This is a classical case of a hard oscillator where galloping is only possible if a large perturbation is applied to excite the system. Such bodies are not ideal for GMPG applications since they are not self excited^26^.
- A gradual reduction in the radius, *R*, of the sides of the bluff body which progressively transforms the top and bottom sides from a line to a curved surface. The non-dimensional value of the curvature, that is *D*/*R* ranges from 0.5 to 1.25. At $D/R = 0.5$, the flow structure around the prism is notably similar to that of the straight shape. Higher values of $D/R > 1.25$ are inadmissible for shapes with high *D*/*d* values.

## Optimization approach

Here, we present the optimization process for finding the optimal bluff body geometry, which goes as follows:

1. Design of experiments (DOE): In this work, Latin Hypercube sampling (LHS)^27^ is used to design the set of experiments covering the input variables space (*D*/*d*, *D*/*R*) across evenly spaced steps of $\alpha = [0,30^o]$ (steps of $2^o$).
2. High fidelity computational model: We run the CFD-model to predict the normal lift force curves (Fig. S1, Supplementary Information). The performance of the model is validated by comparing the results of selected prism shapes with corresponding data from experimental measurements in the literature. To provide the required training data for ANN (surrogate model), each shape arising from the DOE is simulated using the same initial and boundary conditions.
3. Machine learning: We train the ANN based on the DOE data set (i.e. $C_N-\alpha$ curves for different (*D*/*d*, *D*/*R*) cases). In contrast to typical practice, we determine an optimum ANN architecture using genetic algorithm (GA), which ensures accurate predictions of the $C_N$ values with the least error (Supplementary Information, Fig. S2). The trained ANN is then employed to predict the normal lift force $C_N$ for a new, unseen input data sets of $(D/d, D/R, \alpha )$.
4. Identifying the optimal shape: A simple brute force optimization is carried using the ANN model over the design variable space (*D*/*d*, *D*/*R*).
5. Obtaining the power: The corresponding power is then calculated. Optimal power is obtained for bluff bodies subjected to 1-COSINE gust model (“Methods”, Eq. (8)) with varying gust duration(s). The raw energy imparted by the fluid on the oscillating body is proportional to the integral of the velocity square of the oscillating body as follows:
  4 $$\begin{aligned} P =\frac{1}{T}\int _{0}^{T} C\;\dot{Y}^2\;dY, \end{aligned}$$
  where *T* is the total time of oscillations. The energy harvested by a GPMG through a transduction element is lower than the raw power due to the inherent losses introduced by other transmission stages. Nevertheless, when comparing two generators with different cross-sectional shapes operating under similar conditions, Eq. (4) is sufficient to determine the superior device.

**Figure 4:**
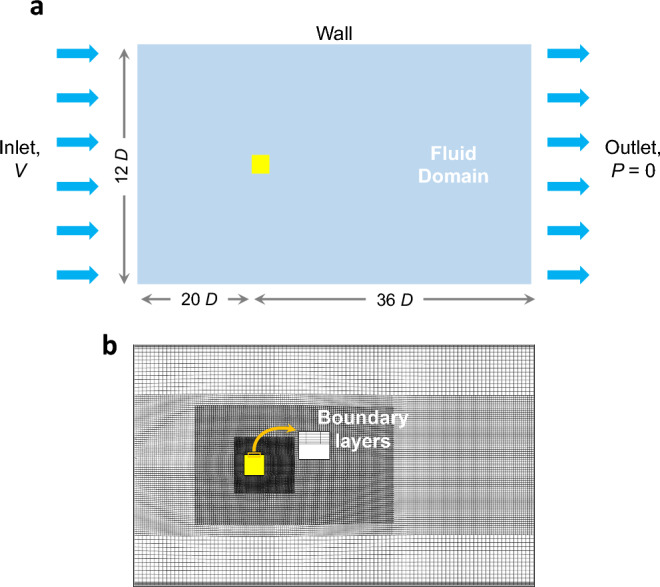
(**a**) Illustration of the computational domain used in the present study, (**b**) mesh and grid topology. The inset shows the boundary layers near the cylinder walls.

## A high fidelity computational model

To model the fluid-structure interactions, we implement a transition model based on Unsteady Reynolds-Averaged Navier-Stokes (URANS) that has the capability to consider the “Laminar-Turbulent” transition in the boundary layer. Laminar boundary layer develops because of increased instabilities of the flow, which accordingly loses its stability and transitions to a turbulent boundary layer. Ordinary URANS modeling cannot capture this phenomenon, and they usually result in fully turbulent boundary layers.

Simulations were performed by the open-source CFD tool OpenFoam^28^ using a pressure-based solver with the finite volume method. Figure 4a depicts the domain of interest, which comprise of a two-dimensional cylinder with a fixed frontal width, *D*, subjected to a uniform free stream velocity, *V*. We consider incompressible flow with constant fluid properties and a fixed Reynolds number of 23, 000 (see details in “Methods”). The inlet is located 20*D* upstream the leading edge of the bluff body, the outlet is located 36*D* downstream the bluff body, and the height is 12*D*. The fluid domain is meshed using hexahedral elements as illustrated in Fig. 4b. The meshed domain consists of 4 distinct regions with varied mesh density. We applied mesh refinement until flow field quantities are mesh independent (see Fig. 12 in “Methods”).

**Figure 5:**
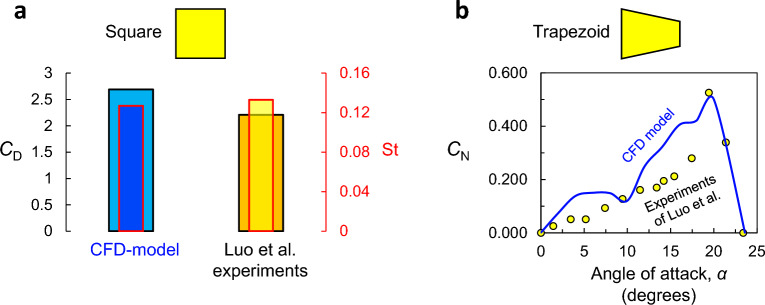
(**a**) Comparison of the time-averaged drag coefficient, $C_D$, and Strouhal number, St, for a square cylinder. (**b**) Normal force coefficient $C_N$ as a function of angle of attack $\alpha$ for a straight trapezoid with side ratio $D/d = 1.\bar{3}$. (circles) represent experimental data points^7^. (line) represent computational data points (CFD model).

The results of the computational model are validated by comparing them to experimental data performed on a square profile, namely the time-averaged drag coefficient, $C_D$, and Strouhal number, $\text {St}$^7^, as shown in Fig. 5a. It can be observed that the results of the CFD model are in good agreement with the independent experiments of Luo et al.^7^, with less than 2.3% error. Nevertheless, this agreement does not indicate whether the computational model is necessarily adequate for other shapes. We therefore investigate the normal force coefficient, $C_N$, for the flow past a trapezoidal prism of $D/d = 1.\bar{3}$ and $D/R = 0$ for angles of attack between ${0}^{\circ }$ and ${25}^{\circ }$. The normal force coefficient can be obtained from the drag and the lift force coefficients using:

5 $$\begin{aligned} C_N(\alpha ) = C_L \cos {\alpha } - C_D \sin {\alpha }. \end{aligned}$$

Figure 5b illustrates variation of the normal force coefficient, $C_N$, with the angle of attack, $\alpha$, and compares it with the experimental data available in Ref.^7^. The CFD-model accurately predicts the maximum value of $C_N^{max} = 0.55$ and the stability angle, $\alpha _{se} = {24}^{\circ }$. The CFD model sometimes overestimates the $C_N$ forces. Despite this small discrepancy, the results clearly showcase the effectiveness of the CFD model in predicting the normal force coefficient for a trapezoidal cross-sections at varying angles of attack.

## Artificial neural network

After validating the CFD model, we use it to generate 800 points of training data: $\bigl ({\textbf {x}}=[\frac{D}{d}, \frac{D}{R}, \alpha ], C_N({\textbf {x}})\bigl )$. We feed the data to an artificial neural network (ANN) training module (see details in “Methods” and Fig. S2a in the Supplementary Information), by which the ANN learns the underlying trends and subtleties of the CFD simulation, specifically in predicting the normal lift coefficient, $C_N$, within the following training bound: ${\textbf {x}}$, between $\frac{D}{d}\in [1,1.66]$, $\frac{D}{R}\in [0.5,1.25]$ and $\alpha \in [{0}^{\circ },{30}^{\circ }]$.

**Figure 6:**
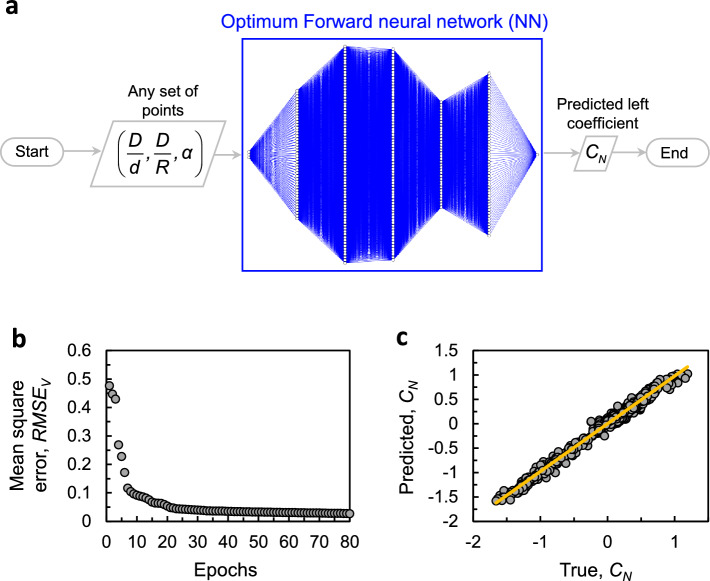
Training the optimum ANN. (**a**) Flow diagram of the training process of final optimum ANN architecture obtained by the genetic algorithm scheme. (**b**) Validation mean square error at every training iteration (epochs). (**c**) Comparison between predictions and the actual validation data set of $C_N$.

As shown in Fig. 6a, the parameter space comprises 3 input variables, also known as the predictors, and one output variable known as the response. Therefore, the ANN consists of 3 input and 1 output neurons, which are fixed. The number of hidden layers, *n*, and the number of neurons within each hidden layer, *i*, are typically adjusted for maximum accuracy and performance. To protect against overfitting, we employ the k-fold validation method^29^ and use a parameter $\gamma$ to measure the regularization strength^30^. This prevents spikes and/or local ripples from appearing in data fitting. The ANN can therefore be generalized by the following array: ${\textbf {N}}=(k, n_1, n_2,..., n_i, \gamma )$.

Often, users of ANNs for regression analysis decide on the number of hidden layers and neurons within using a trial and error scheme, or by relying on previous experience, and/or previously solved cases. Here, we opted to find the ANN parameters $(k, n_1, n_2, ..., n_i, \gamma )$ using a Genetic optimization algorithm (GA, Matlab optimization toolbox^18^). The optimization problem is formally described in supplementary materials, Eq. (1). Running the GA algorithm numerous times yields the same optimum architecture consisting of the following numerical ANN parameters ${\textbf {N}}_f=(10,46,77,75,38,58,0.00088)$. Figure 2 in the supplementary material demonstrates the flow diagram of the genetic algorithm and its interaction with the ANN training module that is outlined in grey.

Figure 6a depicts the final optimum ANN architecture and shows how this ANN will be used to surrogate the CFD predictions of $C_N$. Finally, we train the ANN, where the weights and biases of its layers and neurons are adjusted iteratively to minimize $RSME_V$ using gradient descent method. Figure 6b shows the validation error at every backpropagation iteration (commonly known as the epochs). The validation error decays rapidly, showing how further iterations do not bring about further reduction in the $RSME_V$. Finally, Fig. 6c compares the true with the predicted $C_N$ values. There is a clear agreement between the prediction of ANN and the actual validation set, resulting in a coefficient of determination, $R^2=0.986$. We can now simply feed the parameters, ${\textbf {x}}$ to the ANN and readily predict the respective $C_N({\textbf {x}})$ values.

## Results and discussion

### Effect of geometry on $C_N$

**Figure 7:**
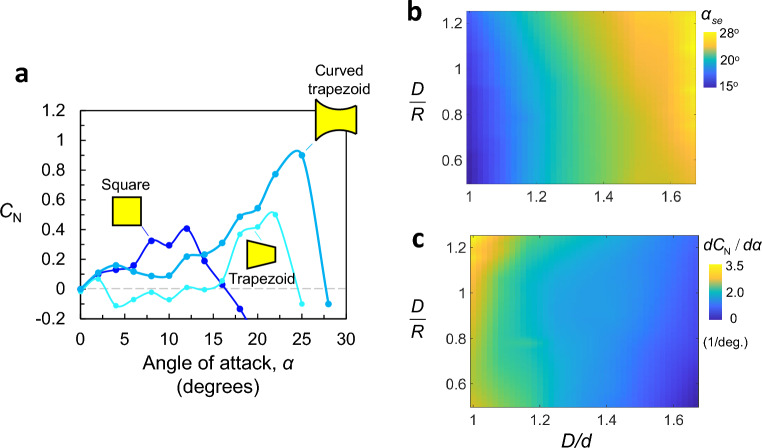
(**a**) Normal force coefficient $C_N$ as a function of angle of attack $\alpha$ for a straight square (*D*/*d* = 1, *D*/*R* = 0), a straight trapezoid (*D*/*d* = 2, *D*/*R* = 0), and a curved-trapezoid (*D*/*d* = 2, *D*/*R* = 1). Maps of (**b**) the stability angle, $\alpha _{se}$, and (**c**) the initial slope of the normal lift curve across the design space of (*D*/*d*, *D*/*R*).

We start by examining three contrasting cross section geometries: a straight square prism (*D*/*d* = 1, *D*/*R* = 0), a straight trapezoid cross-section with no curvature (*D*/*d* = 2, *D*/*R* = 0), and a curved-trapezoid section (*D*/*d* = 2, *D*/*R* = 1) (Note that we deliberately included values beyond $D/d=1.66$ in this initial analysis in order to clearly demonstrate different characteristics between the curves.). The objective of this analysis is to reveal the influence of the side ratio, *D*/*d*, and curvature, *D*/*R*, on the $C_N$ curves. The resulting $C_N$ curves are depicted in Fig. 7a.

The normal lift force of the square prism demonstrates a progressive rise in $C_N$ up to 0.45 between ${0}^{\circ }<\alpha <{12}^{\circ }$. Beyond ${12}^{\circ }$, $C_N$ rapidly decreases to zero. For the straight trapezoid prism, the normal force fluctuates around zero until $\alpha ={15}^{\circ }$, which is akin to classical hard-oscillators. Subsequently, $C_N$ increases to a maximum value of 0.52 between ${8}^{\circ }<\alpha <{23}^{\circ }$, before rapidly dropping with increased $\alpha$. Conversely, for the curved-trapezoid, the normal lift increases up to 0.15 then decreases slightly before reversing trend near ${10}^{\circ }$. The $C_N$ values then rise progressively up to 0.9 before dropping rapidly at an angle ($\alpha \approx 26$).

As stated earlier, two parameters on those curves are quiet important for the performance of GMPGs. The initial slope of the curve, $\frac{dC_N}{d\alpha }$ which correlates directly with the rise time, $\bar{T}$, and the value of the stability angle $\alpha _{se}$ which correlates directly with the steady-state amplitude, $\bar{A}$, (Fig. 2).

In order to visualize the effect of the side ratio (*D*/*d*), and the curvature (*D*/*R*) on both the initial slope and the stability angle, we generated, using the ANN model a fine contour field map of $\frac{dC_N}{d\alpha }$, and $\alpha _{se}$ as function of (*D*/*d*, *D*/*R*) (see Fig. 7b, c). Shapes with a high *D*/*d* tend to have the highest $\alpha _{se}$ between ${24}^{\circ }$ and ${28}^{\circ }$, but the lowest initial slopes. On the other hand, shapes with a low *D*/*d* yield the least stability angles lying between ${15}^{\circ }-{18.3}^{\circ }$, but the highest initial slopes in the range between 2-3.5. Increased curvature (higher *D*/*R*) increases both $\alpha _{se}$ and $\frac{dC_N}{d\alpha }$. The maximum values of the initial slope and the stability angle occur therefore at the highest curvature of *D*/*R* = 1.25.

### Effect of $C_N$ on galloping

**Figure 8:**
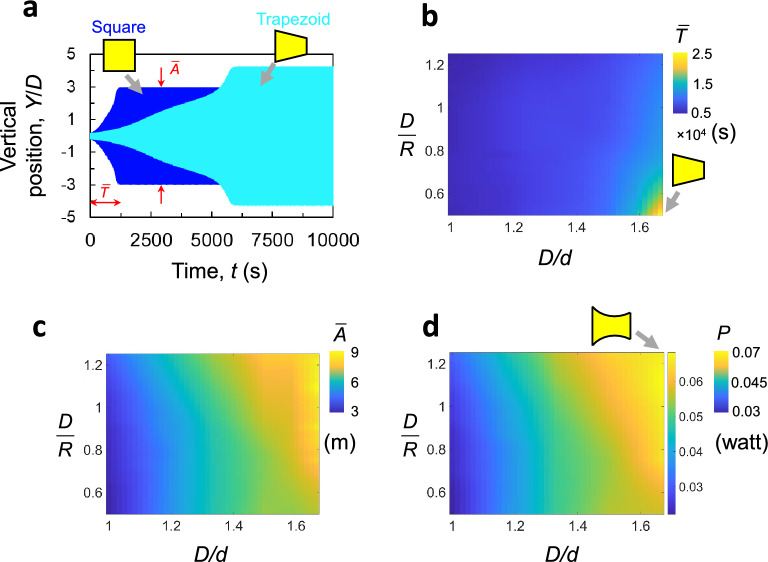
(**a**) Time history of the response amplitude for a square and a straight trapezoid ($D/d = 1.\bar{3}$). (**b**–**d**) contour field map showing the rise time $\bar{T}$ and steady-state amplitude $\bar{A}$, and the resulting power *P*, respectively, across continuum of different *D*/*d* and *D*/*R*.

Next, we investigate the effect of the $C_N$ curve on the galloping behavior of the prisms by performing a numerical integration of Eq. (1) using the $C_N-\alpha$ curves obtained via the ANN model. We initially examine two shapes without curvature: the straight square and the straight trapezoid having $D/d=1.\bar{3}$. Our simulations consider the same parameters listed in Table 1 in the “Methods” section. The aerodynamic coefficients (i.e. $A_j$) are obtained by fitting the $C_N-\alpha$ curve into a polynomial function (Eq. 2). Time histories of the galloping response are presented in Fig. 8a. Recall that the square shape has a steep initial slope $\frac{dC_N}{d\alpha }$ and the lowest stability angle, $\alpha _{se}$. Thus, it has the shortest rise time, $\bar{T}$, to the steady-state value, but the smallest steady-state amplitude, $\bar{A}$. On the other hand, the straight trapezoid has a lower $\frac{dC_N}{d\alpha }$ and a higher $\alpha _{se}$, thus, results in a higher $\bar{T}$ and $\bar{A}$ than that of the square geometry.

Subsequently, we used the predicted curves in conjunction with Eq. (1) to compute the corresponding rise time, $\bar{T}(D/d, D/R)$ and the steady-state amplitude, $\bar{A}(D/d, D/R)$. Figure 8b,c show the contour field maps of those variables. Shapes with high *D*/*d* values, like the curved- and straight trapezoids, exhibit the highest $\bar{A}$ and $\bar{T}$ values. Whereas, prisms with with low *D*/*d*, like the square result in the lowest $\bar{T}$ and $\bar{A}$. Increasing the curvature serves to increase the steady-state amplitude and decrease the rise time, which is most pronounced in the curved-trapezoid case (see top right corners of Fig. 8b,c).

### Power generation

Using Eq. (4), we first compute the power generated under steady wind condition as shown in Fig. 8d for a steady wind speed of $V=10$ $\hbox {m}/\hbox {s}$. It can be clearly seen that the harnessed power increases as either *D*/*R* or *D*/*d* increase and that highest power levels are achieved for the trapezoidal shape with the highest surface curvature and ratio between the frontal and dorsal widths. Conversely, the square prism results in the lowest power levels under steady conditions.

**Figure 9:**
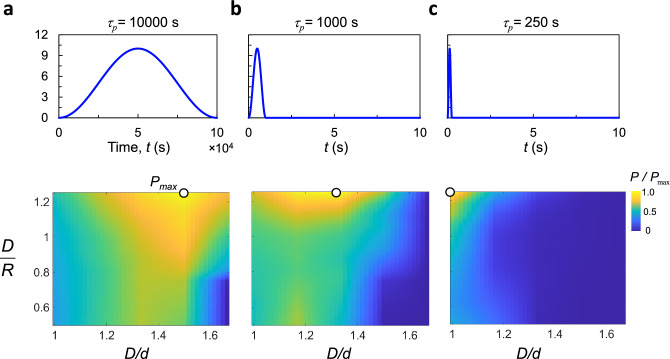
Contour field map showing the power contained in various gust idealizations. (**a**) $\tau = 10{,}000$ s, (**b**) $\tau = 1000$ s, and (**c**) $\tau = 250$ s.

In realistic environments where wind is unsteady, both transient and steady-state response are critical factors for power generation. Thus, the effect of various gust properties on the optimal shape of the GMPG must be analyzed. To this end, we generate power field contour maps for three gust profiles as shown Fig. 9 (see “Methods” section for more details). Long gust profiles (Fig. 9a) generate the highest power output for cases with high *D*/*d* and high *D*/*R* (*P*=0.046 Watt). This is similar to the steady-state case. Despite the long gust period, the power output for high *D*/*d* and low *D*/*R* (straight trapezoid) are negligible. Recall that Fig. 8b, showed how such trapezoid have an extremely long rise time, resulting in a significant decrease in power output. When the gust duration is decreased to $\tau = 1000$ s, we observe a shift in the maximum power extraction towards prisms with lower *D*/*d* values; i.e. shapes that have higher rise time as shown in Fig. 9b. For extremely fast wind fluctuations, where gust durations are extremely small, the rise time becomes the factor dominating power extraction. For instance, as shown in Fig. 9c, the maximum power is achieved when using the lowest value of *D*/*d*. We may therefore conclude that the optimal geometry for power generation is not only dependent on the geometry of the bluff body, but also on the flow conditions. Under more fluctuating winds, the curved-square case with highest curvature performs better, while for steady wind conditions, the curved-trapezoid with high *D*/*d* and *D*/*R* performs the best.

## Flow field analysis

In this section, we study the flow field around the same prisms of Fig. 7a. As mentioned earlier, the performance of GMPGs is strongly correlated with the interaction between the separated shear layers and the side faces. Therefore, mean velocity streamlines can offer an insight into why certain profiles perform better than others.

**Figure 10:**
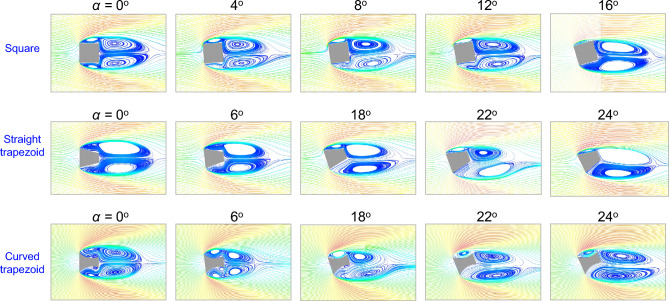
Mean velocity streamlines at different angles of attack.

We start by examining the flow around the square bluff body as shown in Fig. 10. Flow separation occurs at the front corners forming two shear layers that trap two shallow recirculation regions above and below the side faces. At zero angle of attack, the flow pattern is symmetric around and behind the bluff body where two large re-circulation regions appear. The end of the separated wake is indicated by a saddle point in the streamlines. As the angle of attack increases to $\alpha$ = ${4}^{\circ }$, the separated region extends. At the leeward surface relative to the flow, the circulation region decreases in size and moves away from the side face. Conversely, on the windward surface, the side circulation bubble moves closer to the surface of the body. The presence of circulation bubbles entrains the fluid causing higher suction pressure. As such, the normal lift force will be positive. When $\alpha$ = ${8}^{\circ }$, the lower side and the leeward recirculation regions gradually merge, while the recirculation on the windward side moves closer to the prism’s surface. At $\alpha$ = ${12}^{\circ }$, the shear layer reattaches to the side surface, resulting in the highest suction pressure, and thus highest normal force coefficient. As the angle continues to increase past the reattachment angle, the size of the windward circulation bubble decreases.

Variation in the behavior of the shear layer between angles ${12}^{\circ }$ and ${16}^{\circ }$ does not lead to a significant change in the flow characteristics, but it causes a significant reduction in the normal lift force, which goes from a peak value of 0.45 to zero as shown in Fig. 7a. This is due to the reduction in the lift force and the increase in the drag force. The reduction in lift is a result of the decrease in the size of the windward circulation bubble, while the increase in drag is due to the leeward circulation bubble moving closer to the body and causing higher negative pressure. The reduction in lift and increase in drag, which occur right after the reattachement angle contribute to the sudden drop in the normal lift force as per Eq. (5).

The mean flow streamlines around the straight trapezoid ($D/d = 2$) are depicted in Fig. 10. Despite the differences in the afterbody shape between the square and the trapezoid, the flow patterns closely resemble that of the square. For instance, at $\alpha = {0}^{\circ }$, the flow is predominantly symmetrical. When the angle of attack increases beyond ${18}^{\circ }$, the windward circulation bubble increases in size and moves closer to the body, while the leeward circulation bubble merges with the rear wake bubble. At $\alpha$ = ${22}^{\circ }$, the upper shear layer reattaches to the upper surface, leading to the highest lift force, and the rear wake bubble is seen to extend causing lower drag forces. Beyond the re-attachment angle, the windward circulation bubble decreases in size and the lower rear bubble moves closer to the rear face, causing the normal lift force to drop rapidly from 0.52 to 0 (see Fig. 7a). The differences between the square and the straight trapezoid are more pronounced at lower angles of attack. Despite the asymmetry in the flow field for the trapezoid at ${6}^{\circ }$, the windward side face is still relatively far away from the separated shear layers, and thus, no circulation bubbles form. As a result, $C_N$ remains at zero until the windward bubble appears (see Fig. 7a).

Next the flow field for the curved trapezoid ($D/d = 2$, $D/R = 1$) is explored with the goal of understanding the role of the curvature on the flow patterns. Unlike the non-curved trapezoid and due to curvature of the side faces, leading edge recirculation bubbles are present even at zero angle of attack. Furthermore, two new secondary bubbles form near the rear corner of the side faces. As the angle of attack increases to ${6}^{\circ }$, the symmetry between the bubbles is disrupted, which leads to a differential pressure between the upper and lower surfaces causing a net lift on the body, as demonstrated by the positive initial slope of the $C_N$ curve in Fig. 7a. As the angle increases further to ${18}^{\circ }$, the windward primary bubble merges with the secondary bubble forming a larger bubble, which causes an increase in suction pressure and a large positive net lift. Yet, on the leeward surface relative to the flow, the primary bubble merges with the wake bubble extending it further downstream. At $\alpha$ = ${22}^{\circ }$, two main flow patterns contribute to the remarkable normal lift force seen in Fig. 7a. First, the leeward secondary circulation bubble disappears, reducing the suction pressure on the lower surface, thereby increasing the positive net lift. Second, the rear wake bubble extends further downstream of the prism, leading to a decrease in the drag, thereby increasing the overall normal force. At $\alpha$ = ${24}^{\circ }$, the curvature maintains a relatively large upper circulation bubble when compared to that of the straight trapezoid at the same angle of attack. Furthermore, the interaction between the shear layers preserves the increased wake formation length. As a result, the normal lift force continues to grow to a value of 0.9 as shown in Fig. 7a. As the angle continues to increase beyond $\alpha$ = ${26}^{\circ }$, the windward circulation bubble decreases in size and the lower wake bubble moves closer to the prism’s rear face. As a result, the lift force decreases and the drag force increases causing the $C_N$ to drop significantly.

## Experimental results

**Figure 11:**
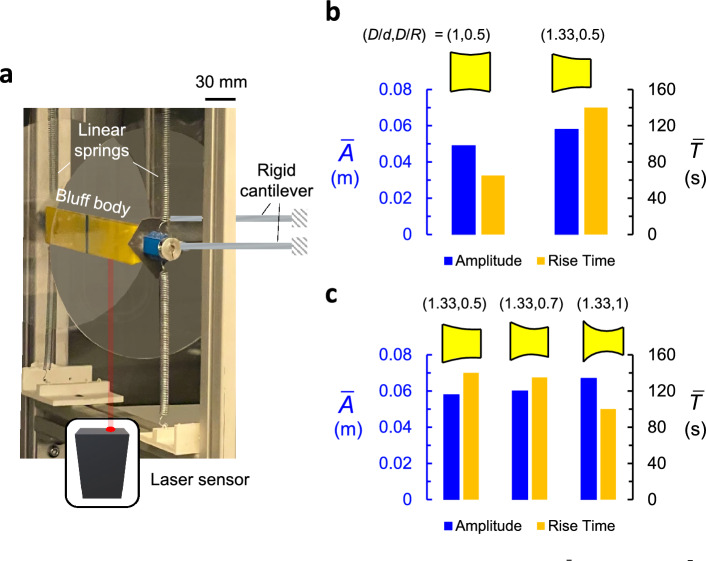
(**a**) Overview of the experimental setup. (**b**) Comparison of the steady-state amplitude $$\bar{A}$$ and the rise time $$\bar{T}$$ for Square 1 ($$D/d = 1 $$, $$D/R = 0.5$$ ) and trapezoid 2 ($$D/d = 1.33 $$, $$D/R = 0.5$$). (**b**) Comparison of the steady-state amplitude $$\bar{A}$$ and the rise time $$\bar{T}$$ for trapezoid 2 ($$D/d = 1.33$$, $$D/R = 0.5 $$), trapezoid 3 ($$D/d = 1.33$$, $$D/R = 0.7 $$), and trapezoid 4 ($$D/d = 1.33$$, $$D/R = 1 $$).

To understand whether the theoretical trends can be replicated experimentally, we investigate the dynamic behaviour of selected bluff body shapes subject to steady wind speed. To this end, galloping bluff bodies of selected shapes were designed, 3D printed, and tested in a wind tunnel to gather data characterizing their transient response. The experimental setup is illustrated in Fig. 11a (see details of the experimental study in the “Methods” section).

The experiments are started while the body is restrained. The flow is then ramped up to the desired flow speed, here chosen as $$V = 5.7$$ $$\hbox {m}/\hbox {s}$$, to be large enough to avoid regions where vortex-induced vibrations can influence the galloping response^31^. Subsequently, the body is released to move and the displacement of the body is recorded. The recording stops when the oscillator achieves a steady-state amplitude. Using the time history of the response, both of the steady-state amplitude and the rise time are recorded. The steady-state amplitude and the rise time are compared for the four different shapes: a curved-square ($$D/d = 1 $$, $$D/R = 0.5$$ ), and curved-trapezoids of ($$D/d = 1.33 $$, $$D/R = 0.5$$), ($$D/d = 1.33$$, $$D/R = 0.7 $$), and ($$D/d = 1.33 $$, $$D/R = 1$$).

The first experiment demonstrates a comparison between the curved square and a slightly-curved trapezoid ($$D/d = 1.33 $$, $$D/R = 0.5$$) as shown in Fig. 11b. Note that, both shapes have the same low radius of curvature, but different side ratios. It can be seen that the results are consistent with the predictions shown earlier in Fig. 8b,c. In particular, the amplitude resulting from the curved-trapezoid, $$A = 0.058$$ $$\hbox {m}$$, is significantly larger than that achieved by the curved-square, $$A = 0.049$$ $$\hbox {m}$$. However, the curved-trapezoid requires almost twice the time to achieve the steady-state amplitude.

The second experiment compares the three shapes having the same side ratio ($D/d=1.33$), but increasing radii of curvature as shown in Fig. 11c. When the curvature of the trapezoid is increased from 0.5 to 0.7, we observe that the steady-state amplitude, $\bar{A}$ increases and that the rise time, $\bar{T}$ decreases. The same trend continues when the radius of curvature is increased further from $D/R=0.7$ to $D/R=1$ as $\bar{A}$ increases by 10 %, and $\bar{T}$ decreases by more than 35 %.

It can be seen that the experimental trends are in qualitative agreement with the aforedescribed predictions of the AI-based optimization. Both demonstrate that increasing the side ratio enhances the steady-state amplitude, but decreases the rise time, while increasing the curvature improves both the steady-state amplitude and the rise time.

## Conclusion

We employed a machine learning prediction algorithm in conjunction with CFD simulations to optimize the shape of the bluff body of GMPGs under steady and transient flow conditions. The machine learning model is designed to overcome the burden of computational resources by building a neural network capable of predicting the normal force coefficient for various shapes with limited training data. Since there are infinitly many body shapes, we limit this study to a continuum shape description, which encapsulates most of the cases studied earlier in the literature. The continuum has a straight frontal and dorsal faces with varying lengths, and side faces described by surfaces of different curvature. The specific conclusions are as following:

- Reducing the rise time and maximizing the steady-state amplitude are competing objectives when changing the frontal-to-dorsal side ratio of the considered shapes. In particular, a larger side ratio is associated with higher rise time but larger steady-state amplitude and vise versa.
- Increasing the curvature of the side surfaces reduces the rise time to the steady-state value while maximizing the steady-state amplitude.
- Under steady flow conditions, the optimal shape for the GPMGs is the one that maximizes the steady-state amplitude regradless of the rise time. This is the curved-trapezoidal shape with the highest side surface curvature and frontal-to-dorsal side ratio.
- Under highly fluctuating flow conditions, the rise time becomes the factor determining the raw power. Specifically, when the duration of the flow gust is short, shapes with faster growth rates produce higher output power. This is the curved-square shape with the highest side surface curvature.
- Apart from those two extreme flow conditions, the optimization algorithm leads to shapes that are somewhere in between the curved-trapezoidal shape with the highest side surface curvature and frontal-to-dorsal side ratio and curved-square shape with the highest side surface curvature.
- By analyzing the flow field around selected prism shapes, we have found that the curvature of surfaces enhances galloping through two distinct mechanisms. First, the curvature produces new circulation bubbles at zero angles of attack. As the angle of attack increases, the symmetry between those bubbles are disrupted causing a differential pressure between the side surfaces leading to an increase in the normal force at small angle of attacks (higher initial slope). Second, the curvature plays an important role towards delaying the stability angle in the $C_N$ curve. In particular, the curvature creates a larger space for the re-circulation bubbles to be maintained, thereby increasing the lift force.
- Using wind tunnel tests, the theoretical trends were replicated experimentally under steady wind conditions.

## Methods

### Parameters

The materials and geometric parameters adopted in the analytical mass-spring-damper model are listed in Table. 1.

### High fidelity computational model details

The simulations were performed by the open-source CFD tool OpenFoam^28^ using a pressure-based solver and the finite volume method. The PIMPLE (Pressure Implicit Method for Pressure-Linked Equations) algorithm was used to handle the pressure and the velocity coupling equations. The K-Omega Shear Stress model (SST) approach, based on the model by Menter^32^ was adopted, and the bounded central differencing was applied to the momentum equations. An implicit scheme with second-order accuracy was applied to the time term.

The computational domain is depicted in Fig. 4a. A two-dimensional cylinder with constant frontal diameter, *D*, is exposed to a constant free stream velocity, *V*. An incompressible flow with constant fluid properties was assumed. The Reynolds number defined by; $Re=\rho V D/\mu$, is set to 23, 000, and the rest of the fixed parameters are $D = 0.035$ $\hbox {m}$, $V= 10$ $\hbox {m}/\hbox {s}$, and $\mu = 1.8E-05$ $\hbox {kg}/\hbox {m s}$.

The computational domain includes four boundary patches: inlet, outlet, side walls, and the cylinder. Neumann boundary conditions were applied on the pressure at the inlet, the pressure at the walls, and the velocity at the outlet. While Dirichlet boundary conditions were applied on the velocity at the inlet, the velocity at the walls, and the pressure at the outlet. We imposed the no-slip condition at the cylinder and side walls. We adopted the K-Omega Shear Stress model (SST) approach as the turbulence model^32^. Particularly, we assumed isotropic turbulence model at the inlet. At the inlet, the initial turbulent kinetic energy, *k*, and turbulence specific dissipation rate, $\omega$, were estimated by assuming an isotropic turbulence model using the following formulae:

6 $$\begin{aligned} k&= \frac{3}{2} \left( T_i V\right) ^2, \quad \quad \omega = \frac{\rho k}{\mu } \left( \frac{\mu _t}{\mu }\right) ^{-1}, \end{aligned}$$

where $T_i$, is the turbulence intensity, which defines the amount of turbulence present at the inlet, here assumed to be 0.5% to resemble a low-turbulence case. The eddy viscosity ratio denoted by $\frac{\mu _t}{\mu }$ is set to 1. At the cylinder, the turbulence kinetic energy, $k = 0$ $\hbox {m}^{2} \hbox {s}^{2}$ was adopted from Ref.^32^. To estimate $\omega$ at the cylinder wall, the following relation is used^33^:

7 $$\begin{aligned} \omega = \frac{6 \mu }{\beta _1 y^2_{wall}}, \end{aligned}$$

where $\beta _1 = 0.075$ and $y_{wall}$ is the wall distance. Details of the initial and boundary conditions are listed in Table 2.

**Table 2:** Initial and boundary conditions.

Variables		Conditions		
	Inlet	Outlet	Walls	Initial Field
*V*	$V_\infty$	$\displaystyle \frac{\partial V}{\partial x} = 0$	$V = (0,0,0)$	$V = (10,0,0)$
*p*	$\displaystyle \frac{\partial p}{\partial x} =0$	$p = 0$	$\displaystyle \frac{\partial p}{\partial x} =0$	$p = 0$
*k*	$k =0.00375$	$\displaystyle \frac{\partial k}{\partial x} =0$	$k = 1E-11$	$k =0.00375$
$\omega$	$\omega =250$	$\displaystyle \frac{\partial \omega }{\partial x} =0$	Wall function	$\omega =250$

### Mesh generation scheme

 An unstructured mesh was utilized for the computational domain as shown in Fig. 4b. The mesh was generated utilizing SnappyHex tool, a hexahedral mesh generator that was controlled through the use of OpenFoam dictionaries. The grid generation process begins by defining the cell height near the cylinder walls. The near wall cell spacing is of paramount importance for accurate prediction of the velocity gradients normal to the wall. Steep gradients in the viscous sublayer require low first cell height ($y+<1$) to capture important flow features such as shear layer separations that directly influence the drag and the lift forces. After defining the cell wall height at the cylinder, an exponential cell size growth was applied to the edges around the cylinder wall. Subsequently, a fine mesh was imposed to capture the expected large fluctuations due to flow separations near the cylinder. Finally, three decreasing mesh density regions were created taking into consideration: (1) orthogonality of the grid, (2) relatively high grid density in regions where steep gradients and large truncation errors are expected, and (3) smooth transition in the grid between the different regions.

**Figure 12:**
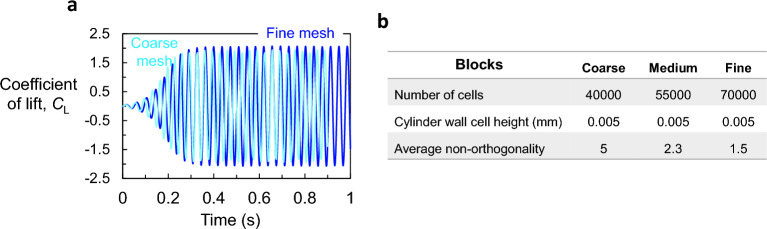
(**a**) Grid sensitivity study. (Solid dark blue line) represents the fine grid, (solid blue line) the medium grid, and (solid cyan line) the coarse grid. (**b**) Table detailing the different mesh densities used the convergence study.

Convergence study was performed to delineate the flow pattern around the stationary square prism. Simulations were carried out for three distinct grids. Figure 12a and b illustrate time history of the lift force coefficient, $C_L$, for the three grids. It can be seen that both the medium grid and the fine grid converge with a relative error of less than 3%. Therefore, the medium grid was adopted for, because it strikes good compromise between computational cost and adequate refinements.

### Details of the artificial neural network

#### Training data generation

The CFD model allows the prediction of the coefficient of lift, $C_N$, for any set of geometric parameters, $C_N = f\bigl ({\textbf {x}}=[\frac{D}{R}, \frac{D}{d}, \alpha ]\bigl )$. One objective of this work is to surrogate this high fidelity CFD model with a forward fully-connected artificial neural network (ANN), that is fast in probing unexplored regions in the design parameter space, ${\textbf {x}}$. To this end, the CFD can be used to generate accurate predictions of, $C_N$, for any set of parameters ${\textbf {x}}$, which can then be used as a training data set to train the ANN predictor. To do so, we first sampled the parameter space using the Latin Hyper Cube Method^34^, which is an efficient design of experiment approach that covers the parameter space within intended bounds with the least number of points (CFD realizations). We sampled a total of 800 points across the 3D parameters space $\bigl (\frac{D}{d}, \frac{D}{R}, \alpha \bigl )$, between $\frac{D}{d}\in [1,1.66]$, $\frac{D}{d}\in [0.5, 1.25]$ and $\alpha \in [0^o,30^o]$, with a resolution of $2^o$. We ran the CFD model for those 800 points and compute corresponding $C_N$ values. Note that each CFD realization requires around 5 h of real time (a total of 0.73 years of simulation time). As such, we utilized a high-performance computing (HPC) cluster to run those computationally expensive CFD realizations.

#### Deep learning

We have 3 design variables, also known as predictors, which are the inputs, together with a single output (known as the response). Therefore, the neural network comprises of 3 input neurons and 1 output neuron which are fixed. The number of hidden layers, *n*, together with the number of neurons within each hidden layers, *i*, are typically adjusted for maximum accuracy and performance. The ANN was trained using backpropagation, where the weights matrices, ${\textbf {W}}$, and biases vectors, ${\textbf {b}}$, that determine the activation of individual neurons within the ANN were iteratively changed to minimize the mean square error against the training data using a gradient decent method known as limited-memory Broyden–Fletcher–Goldfarb–Shanno quasi-Newton algorithm (LBFGS^35^). In addition, we used ReLu as the activation function within the neurons of the hidden layers. We further protected against overfitting by employing the *k*-fold validation method. An additional parameter known as regularization strength was also employed to prevent overfitting. The aforementioned ANN training module steps are summarized as a block diagram in Supplementary Information, Fig. S2.

#### ANN optimization

It is customary to start off with an initial architecture of ANN, that is often set arbitrarily, and sometimes adjusted to achieve a reasonable balance between performance and $RMSE_V$. Several works, have used parametric study analysis over the ANN’s number of hidden layers and neurons for a given problem of interest to achieve minimum $RSME_V$ values. For a more systematic analysis and representation of the CFD simulations, we found the set of ANN parameters that yields the lowest $RMSE_V$ by formulating it as a contrained optimizations problem (Supplementary Information 1). To this end, we utilized the genetic algorithm scheme available in Matlab optimization toolbox^18^.

### Wind gust model

We adopted standard 1-COSINE gust model with varying durations, with temporal profiles. Wind gust profiles can be viewed as a series of isolated pulses and the function that defines the velocity in the time domain is written as:

8 $$\begin{aligned} V = \frac{V_m}{2} \left( 1-\cos {\frac{\pi t}{\tau }}\right) (H(\tau ) - H(2 \tau )), \end{aligned}$$

where $H(\tau )$ is the Heaviside step function, $V_m$ is the peak gust velocity; that is, the maximum amplitude of the 1-COSINE gust shape. The period of the gust is given by $\tau$. Note that, the form presented here is represented by two Heaviside step functions. This enables the numerical simulation to continue even after the gust subsides.

### Experimental setup

Experiments were conducted in an open-circuit wind tunnel. The test section is 0.6 m wide and 0.6 m height. The flow speed can be varied continuously up to 40 m/s. The bluff bodies were 3D printed. End plates were installed to create a two-dimensional flow and to suppress any undesirable stream-wise flow. The mass of the model and the end plates combined is $M = 0.45$ $\hbox {kg}$. The Reynolds number (Re) of the flow ranges between 4455 and 15,600. The bluff body was placed horizontally in the wind tunnel and suspended using four elastic springs, which have a nominal stiffness of 90 $\hbox {N}/\hbox {m}$. Translation of the model in the direction of the wind is restrained by means of two long low stiffness cantilever beams fixed downstream the body. The beams are long enough to minimize rotational effects. A Micro-Epsilon optoNCDT 1750 laser vibrometer was used to measure the amplitude of the displacement. Transverse vibration testing in this basic configuration resulted in a natural frequency of 4.5 $\hbox {Hz}$ and a critical damping ratio of 0.002, see Fig. 11a

### Supplementary Information

Supplementary Information.

## Data Availability

The datasets generated during and/or analysed during the current study are available from the corresponding author on reasonable request.
