# Supplementary material for: AI-based shape optimization of galloping micro-power generators: exploring the benefits of curved surfaces
Source: Sci Rep. 2024 Jan 18;14:1552. doi: 10.1038/s41598-024-51979-8 (PMC10794239; doi:10.1038/s41598-024-51979-8)
Supplement: Supplementary file 1 — Supplementary Information. [file 41598_2024_51979_MOESM1_ESM.pdf]

Supplementary Information

Supplementary Figures **2**

Supplementary Information 1: ANN architecture optimization **4**

Supplementary Figures

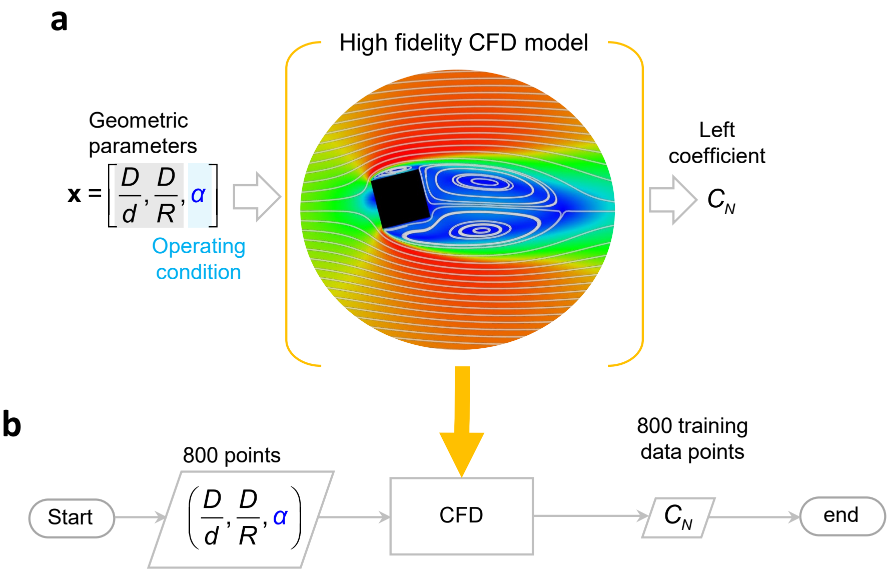

Supplementary Figure 1 Training data generation flow diagram. (a) Depiction of the CFD model. (b) Data generation flow diagram, where parameters, $\mathbf{x}$ are fed into the CFD block and so the C_N values are computed.

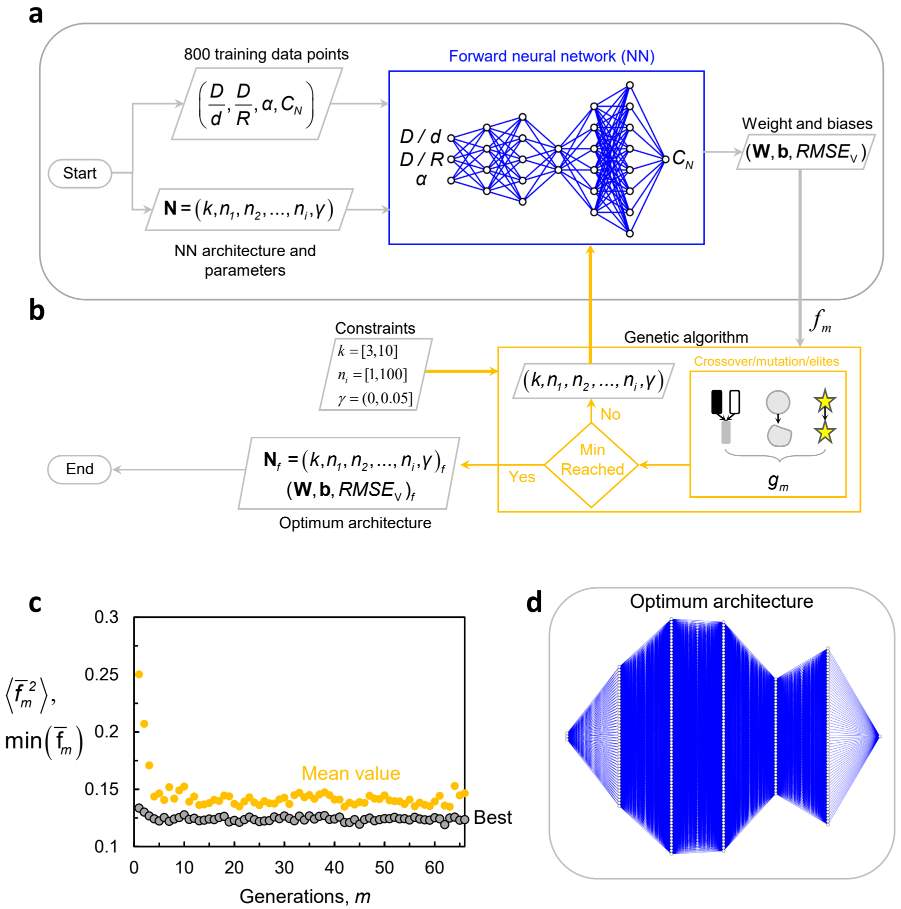

Supplementary Figure 2 Training module and its interaction with Genetic optimization algorithm (GA). (a) The training module, where training data: $\left(\frac{D}{d}, \frac{D}{R}, \alpha, C_N\right)$ along with a neural network parameters: $\mathbf{N}$ to construct corresponding ANN and obtain its trained weight $\mathbf{W}$, biases $\mathbf{b}$, and root mean square error, $f_m = RSME_V$, at every GA iterations denoted by the subscript m. (b) A brief depiction of Genetic algorithm inner workings and requests of training output $(k, n_1, n_2, ..., n_i, \gamma)$ of its newly formed generation at every GA iterations m; (c) The training validation error for every generation, where mean score is indicated in orange while minimum score (best individual within a generation) is indicated in a grey marker with black outline. (d) shows the optimum ANN arrived at by the GA scheme. computed.

Supplementary Information 1: ANN architecture optimization

The parameters which determines the architecture of the ANN are predominantly positive integers (k, n, i). Only the regularization strength which is a continuous parameter; $\gamma \in \mathbb{R}^{+}$. For a more systematic analysis and representation of the CFD simulations, our objective hear is to find the set of ANN parameters $(k, n_1, n_2, ..., n_i, \gamma)$ that yields the lowest $RMSE_V$. Formally, this represents a constrained optimization problem, which reads:

$$
\begin{aligned}
\min \quad & f(k, n_1, n_2, ..., n_i, \gamma) = RSME_V \\
\text{s.t.} \quad & k = [3, 10] \\
& n_i = [1, 100] \\
& \gamma = (0, 0.05]
\end{aligned}
\tag{1}
$$

where, we fixed the number of hidden layers to $i = 5$ layers and $k, n_i \in \mathbb{N}^{+}$. Therefore, ANN design parameters are: $(k, n_1, n_2, n_4, n_5, \gamma)$. The training module described above will be used to evaluate the fitness function f and therewith the respective validation error, $RSME_V$. Bearing in mind that Neural networks in general are a highly nonlinear system due to the nonlinear activation functions and the interplay between layers and neurons, we opted here to use Genetic algorithm for solving Eq.(1).

Figure 2 demonstrates the flow diagram of the genetic algorithm and its interaction with the ANN training module that is outline in grey. The constraints are input to the genetic algorithm first. The algorithm will randomly sample out an initial population (a set of 200 different points of $\mathbf{N}s$ across ANN architecture space), using a creation function(@gacreationuniformmint), those scattered sampled ANN architecture cases are then evaluated (trained) using the training module, $f(\mathbf{N})$ and thus outputs the corresponding set of validation errors $RSME_V$. Those error are scaled by the maximum realization (@fitscalingrank), which resembles the score of individuals, $\bar{f_m}$ within the initial population of the first generation, g_1. The scaled error allows the ranking of the individuals and hence the current m^{th} population gets classified into three groups. A first group of 5% of individuals pass to the next generation as “elites”. The second group undergo mutation (random changes via @mutationpower), while the remaining group is subdivided into ”parents” that crossover among each other (@crossoverlaplace [**?**]). The selection process of parents is based on random duel of 4 individuals at a time across the population, the best performing (lowest f) are selected as “parents” (@selectiontournament). The resulting children, together with the mutated and elite individuals form the next generation. This new generation of new supposedly enhanced ANN architectures are fed back to the training module to get $f(\mathbf{N})$. This process repeats, thereby producing new generations at every iteration g_m, until either one of the stopping criteria is reached. Here the algorithm stops until the average relative change in $f(\mathbf{N})$ is less than a tolerance of

10^{-7}. Once this stopping criterion is satisfied, the algorithm outputs a final optimum architecture: $\mathbf{N}_f = (k, n_1, n_2, n_3, n_4, n_5, \gamma)_f$ along with its respective trained weights and biases: $(\mathbf{W}, \mathbf{b}, RSME_V)_f$, retrieved from the training module. Figure 2c shows the minimum, $\min(\bar{f}_m)$ and mean scaled scores, $\langle f_m \rangle$ of each generation during genetic evolution process. The mean values are indicated in orange while the minimum scores (i.e. best cases) are indicated by grey markers. To the right of Figure 2d, the optimum architecture having; $\mathbf{N}_f = (10, 46, 77, 75, 38, 58, 0.00088)$ is depicted.
